# Development of a Highly Proliferated Bilayer Coating on 316L Stainless Steel Implants

**DOI:** 10.3390/polym12051022

**Published:** 2020-05-01

**Authors:** Fatemeh Khosravi, Saied Nouri Khorasani, Shahla Khalili, Rasoul Esmaeely Neisiany, Erfan Rezvani Ghomi, Fatemeh Ejeian, Oisik Das, Mohammad Hossein Nasr-Esfahani

**Affiliations:** 1 Department of Chemical Engineering, Isfahan University of Technology, Isfahan 8415683111, Iran; f.khosravi@ce.iut.ac.ir (F.K.); shahla.khalili65@gmail.com (S.K.); 2 Department of Materials and Polymer Engineering, Faculty of Engineering, Hakim Sabzevari University, Sabzevar 9617976487, Iran; 3 Department of Mechanical Engineering, Center for Nanofibers and Nanotechnology, National University of Singapore, Singapore 119260, Singapore; erfanrezvani@u.nus.edu; 4 Department of Cellular Biotechnology, Cell Science Research Center, Royan Institute for Biotechnology, ACECR, Isfahan 8159358686, Iran; fatemeh.eje@gmail.com; 5 Material Science Division, Department of Engineering Sciences and Mathematics, Luleå University of Technology, 97187 Luleå, Sweden

**Keywords:** biocomposites, nanofibers, electrospinning, cell culture, graphene oxide

## Abstract

In this research, a bilayer coating has been applied on the surface of 316 L stainless steel (316LSS) to provide highly proliferated metallic implants for bone regeneration. The first layer was prepared using electrophoretic deposition of graphene oxide (GO), while the top layer was coated utilizing electrospinning of poly (ε-caprolactone) (PCL)/gelatin (Ge)/forsterite solutions. The morphology, porosity, wettability, biodegradability, bioactivity, cell attachment and cell viability of the prepared coatings were evaluated. The Field Emission Scanning Electron Microscopy (FESEM) results revealed the formation of uniform, continuous, and bead-free nanofibers. The Energy Dispersive X-ray (EDS) results confirmed well-distributed forsterite nanoparticles in the structure of the top coating. The porosity of the electrospun nanofibers was found to be above 70%. The water contact angle measurements indicated an improvement in the wettability of the coating by increasing the amount of nanoparticles. Furthermore, the electrospun nanofibers containing 1 and 3 wt.% of forsterite nanoparticles showed significant bioactivity after soaking in the simulated body fluid (SBF) solution for 21 days. In addition, to investigate the in vitro analysis, the MG-63 cells were cultured on the PCL/Ge/forsterite and GO-PCL/Ge/forsterite coatings. The results confirmed an excellent cell adhesion along with considerable cell growth and proliferation. It should be also noted that the existence of the forsterite nanoparticles and the GO layer substantially enhanced the cell proliferation of the coatings.

## 1. Introduction

At present, numerous types of bone diseases, e.g., bone fractures, bone infections, bone cancers, and genetic diseases are rising due to increasing prevalence of physical inactivity, obesity and lack of safe exercising [1]. It is reported that over 20 million people suffer from bone disorders and clinical troubles annually, making this an global issue [2]. Traditional bone regeneration methods were based on utilizing autograft and allograft [3]. There are serious drawbacks for using bone substituents from the patient’s iliac crest, including limited donor tissue, donor site illness and increased risk for infections or disease transmission, which highlights the importance of engineered implants [1,4]. Emerging tissue engineering strategies provide a remarkable opportunity for the regeneration of injured tissues through the fabrication of the artificial constructs [5,6]. Such structures must afford a suitable microenvironment for cell attachment and proliferation to stimulate the damaged tissue formation [7]. Furthermore, biocompatibility, biodegradability, and porosity of the structures directly affect their treatment performance [8]. Currently, different types of materials such as metals, polymers, and ceramics are used as biomedical implants [9]. The metallic implants such as stainless steel, cobalt, and titanium alloys are mainly exploited due to the excellent mechanical properties and superior corrosion resistance in orthopedic targets, while polymer and ceramic-based implants exhibit weak and brittle properties [10]. Among the different types of metallic implants, the surgical grade 316LSS is the most common bone-implant offering high mechanical properties, low cost, and availability [11]. Regarding the 316LSS properties, the biggest drawback is the release of the metal ions, e.g., iron, nickel, and chromium in the biological environment, making it pernicious in nature [12,13].

In order to overcome the aforementioned issue, several surface modification procedures have been applied. Based on the literature, the composite coatings method using polymers and ceramic components is considered as the most popular strategy for this purpose [14,15]. Poly (ε-caprolactone) (PCL) is a well-known synthetic polymer composed of semi-crystalline linear polyester, which is approved by the U.S. Food and Drug Administration (FDA) as a biomedical material [16]. Although PCL exhibits significant mechanical strength and biocompatibility, it is inherently hydrophobic which negatively affects its biological properties such as cell adhesion and proliferation [17,18]. Therefore, a combination of PCL with a natural hydrophilic polymer such as Ge was utilized as an ideal coating for bone regeneration [19,20,21]. Ge has been utilized widely in medical applications as a natural biopolymer derived from partial hydrolysis of collagen. In addition to its biocompatibility, low cost, availability the suitable hydrophilicity of Ge-based materials promote cell attachment and proliferation of the blends comprised of Ge [22].

Since rapid biodegradability and weak mechanical properties are considered as the key drawbacks of Ge, it is normally used for tissue engineering combined with artificial polymers such as PCL to fulfill the mechanical properties requirement [23,24]. Yao et al. [25] fabricated PCL/Ge nanofibrous scaffolds containing various polymer ratios for tissue engineering application. The essays of cellular behaviors indicated that the blend of PCL/Ge had higher adhesion and proliferation in comparison with pure PCL and Ge. Additionally, the PCL/Ge having the ratio of 2:1 showed the best cell spreading, viability and cytoskeleton organization. Fanaee et al. [26] prepared PCL/Ge nanofiber mats with a 70/30 weight ratio containing bioactive glass particles via electrospinning for bone tissue engineering application. The results of in vitro tests confirmed no considerable cytotoxicity as well as good cell adhesion for the prepared nanofibers comprised of PCL and Ge. Moreover, bioceramics are exploited to generate osteocunductive feature for these artificial constructs. Various types of bioceramics and bioglasses such as HA, alumina, zirconia, phosphates, and forsterite have been used to stimulate cell growth and/or bone cell formations by releasing active ions in cell microenvironment [27,28,29,30,31]. Recently, forsterite (Mg_2_SiO_4_) has been highly recommended as an osteocunductive biomaterial for use in bone regeneration applications, based on its remarkable mechanical properties and biocompatibility. It is worthwhile to note that forsterite enhances cell proliferation and bone regeneration by releasing Mg ion after implantation. Moreover, a higher degradation rate of forsterite composite scaffolds is reported because of its low degree of crystallinity [32,33]. 

GO is one of the most efficient derivatives of graphene, which has abundant hydroxyls, epoxides and carboxyl functional groups on its surface [34,35]. GO possesses many benefits such as solubility in water and some polar solvents, excellent biocompatibility, good mechanical properties, and high flexibility. It is also a potential biomaterial for cell proliferation enhancement because of its superior biocompatibility. GO nanosheets were incorporated into PCL nanofibers in order to investigate cell behavior of two types of cells such as mMSCs and PC12-L on the PCL/GO [36]. The results showed that GO incorporation substantially improved the cell attachment, spreading and proliferation of the prepared scaffolds. Therefore, the shortcomings of 316LSS—which include releasing of ions such as nickel and chromium—can be addressed using these two materials to improve the biocompatibility and corrosion resistance of 316LSS.

In our previous research, a bilayer coating of GO and polymeric nanofibrous composite was prepared via electrophoretic deposition (EPD) and electrospinning, whereby the corrosion resistance of 316LSS significantly improved [37]. The central aim of this research is evaluating the cellular behavior of that nanocomposite and the bilayer coating. In other words, the effects of GO layer and forsterite concentration on the bioactivity of the nanofibers were assessed.

## 2. Materials and Methods

### 2.1. Surface and Coatings Preparation

The 316LSS substrate was cut into rectangular samples with a dimension of 2 × 1 × 0.4 cm^3^. Before the EPD process, the samples were mechanically polished using SiC papers with 80, 120, 240 and 320 grit-size. Then, the samples were rinsed with deionized (DI) water and were sonicated in acetone to remove any remained grease on the surface of the samples followed by drying at room temperature.

To apply GO coating on the surface of the samples, firstly different amounts of GO nanopowder (Nanosany Corporation, Mashhad, Iran) were dispersed in DI water by ultra-sonication to obtain a homogenous suspension. To reach a uniform coating, different EPD variables such as voltage and deposition time were optimized as discussed in our previous study [37] 

To perform the electrospinning process, solutions containing PCL (average Mw = 80,000, Sigma, St. Louis, MO, USA), gelatin (type B bovine skin, Mw = 50,000–100,000) with 1 and 3 weight percent of forsterite nanoparticles were prepared using formic acid and acetic acid (1:3 *v*/*v*) as solvents. In order to prepare the solvents, forsterite nanoparticles were first ultrasonically dispersed in adequate solvents. Afterward, the solutions were prepared by dissolving the PCL and gelatin in the solvents and magnetically stirred at room temperature for more than six hours. The solutions were then electrospun with a constant gap distance of 15 cm, applied voltage range of 12–26 kV, and feed rates of 0.1–0.5 mL/h. 

### 2.2. Characterization of the Nanofibrous Layer

The morphology of the electrospun samples and distribution of the nanoparticles in the PCL/gelatin nanofibers were evaluated by FESEM (Quanta 450 FEG, Graz, Austria) and Energy Dispersive X-ray (EDS, Octane Elite EDS, Graz, Austria), respectively.

The porosity of the electrospun layer was determined based on the analysis of the nanofiber FESEM micrographs, utilizing image J software (Image J, National Institutes of Health, Bethesda, MD, USA). The surface area of pores (*S_p_*) and the total surface area of the samples (*S_t_*) were measured. Moreover, the porosity percent was calculated according to Equation (1) [38]:

(1) $$\%P=\frac{S_{p}}{S_{t}}\times 100$$

Brookfield DV-II viscometer (Middleboro, MA, USA) and JENWA 3540 conductivity meter (Burlington, NJ, USA) were used to measure the conductivity and viscosity of the electrospinning solutions, respectively. The viscosity was measured at 25 °C and the rotational speed of 6 rpm. Water contact angle measurements were carried out with a drop shape analyzer (Sessile Drop-G10, Tehran, Iran) to investigate the surface wettability and hydrophilicity of the GO layer and electrospun nanofibers. 

The degradation rate of the samples was determined by measuring the weight loss of the samples based on ASTM-F1635 after 21 days of immersion in PBS at 37 °C and pH = 7.4. The weight loss percentage was calculated according to Equation (2). In the equation, the *W*_0_, *W_t_* refer to the weights of the coated samples before and after immersion, respectively. In addition, *W_s_* is the weight of the 316LSS substrate.

(2) $$\%\mathrm{Weight}\;\mathrm{loss}=\frac{w_{0}-w_{t}}{w_{0}-w_{s}}\times 100$$

Since the pH changes indicate the release of the alkaline ions and HA formation [39], the pH value of the solutions was measured during the soaking time using an electrolyte-type pH meter.

### 2.3. Bioactivity Investigations of the Coatings

The bioactivity of the coatings was investigated according to the amount of HA formed on the substrates after soaking in SBF. The SBF solution was prepared according to the Kokubo et al. method [40]. The substrates were immersed in SBF at 37 °C in a stable water bath for 21 days. X-ray diffraction (ASENWARE, AW-XDM 300, Shenzhen, China), using monochromatized CuKα radiation generated from 40 kV and 30 mA and ranging from 10° to 80°, was employed to confirm the crystalline phase of the formed HA on the coated substrates. The morphology of the HA was evaluated by FESEM images after 3, 7, 14 and 21 days soaking in the SBF.

### 2.4. In-vitro Cell Behavior of the Coatings

The MG-63 cells were cultured in Dulbecco’s modified Eagle’s medium (DMEM) complemented with 10% FBS (Gibco, Biosciences, Dublin, Ireland), 1% Glutamax, 1% penicillin/streptomycin and 1% Non-essential Amino Acid (NEAA). The seeded cells were incubated at 37 °C and carbon dioxide amount of 5%. The nanofibrous coatings were electrospun on circular disks based on previous work [41]. All the coatings were sterilized under UV over 15 min on each side, immersed in 70% ethanol for 12 h and then washed with amphotericin/gentamicin/penicillin and PBS for 15 min. After that, the electrospun coated substrates were placed in 24-well plates and MG-63 cells, at a density of 30,000 cells, were seeded on the surfaces of each sample. The cell morphology and adhesion on the seeded nanofibers were evaluated by FESEM images after 1 and 7 days of cell seeding. The cultured cells on the coatings were fixed by 2.5 v% glutaraldehyde solution (Sigma, St. Louis, MO, USA) in PBS and dehydrated through various concentrations of ethanol (0, 25, 50, 75 and 100 v%).

The MTS tests were also performed on the coatings after 1, 3, 5 and 7 days’ cell seeding to evaluate the viability of the samples. The seeded samples were washed and placed in an incubator with 10% of MTS reagent under 37 °C and 5% carbon dioxide. After 3.5 h of incubation, the aliquots were transferred into a 96-well plate. Then, the absorbance of the samples at 429 nm was quantified using a spectrophotometric plate reader (Awareness Technology Inc., Palm City, FL, USA). 

## 3. Results and Discussion

### 3.1. Characterization of the Electrospun Nanofibers

**Figure 1:**
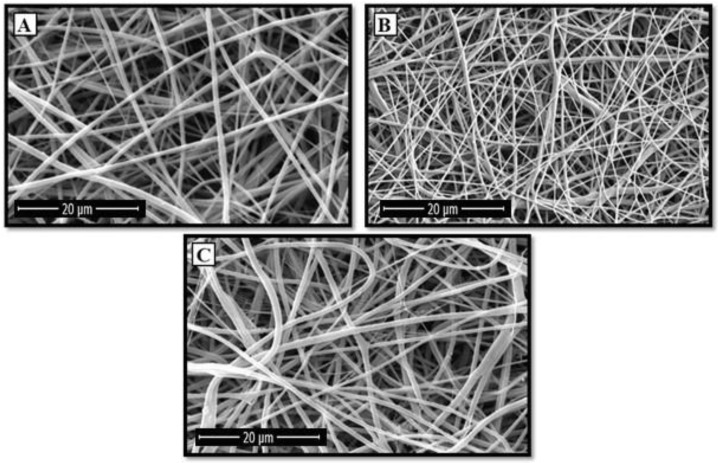
FESEM micrographs of the prepared PCL/Ge nanofibers containing (**A**) 0%, (**B**) 1%, and (**C**) 3 wt.% forsterite nanoparticles.

Figure 1 indicates the FESEM micrographs of the electrospun nanofibers. In addition, the viscosity and conductivity of the solutions along with the average fiber diameter of the electrospun nanofibers were measured and summarized in Table 1 and Table 2, respectively. Table 2 reveals that the incorporation of 1 wt.% forsterite to the PCL/Ge composition decreased the fiber diameter from 167 to 148 nm. Increasing the forsterite content to 3 wt.% increased the average fiber diameter to 171 nm. The addition of the forsterite nanopowder increased the conductivity and the surface charge density of the solution, which caused the diameter reduction. On the other hand, the higher amount of forsterite had a dominant effect on the solution viscosity, leading to an increase in the nanofiber diameter. These results are in agreement with previous researches [42,43].

**Figure 2:**
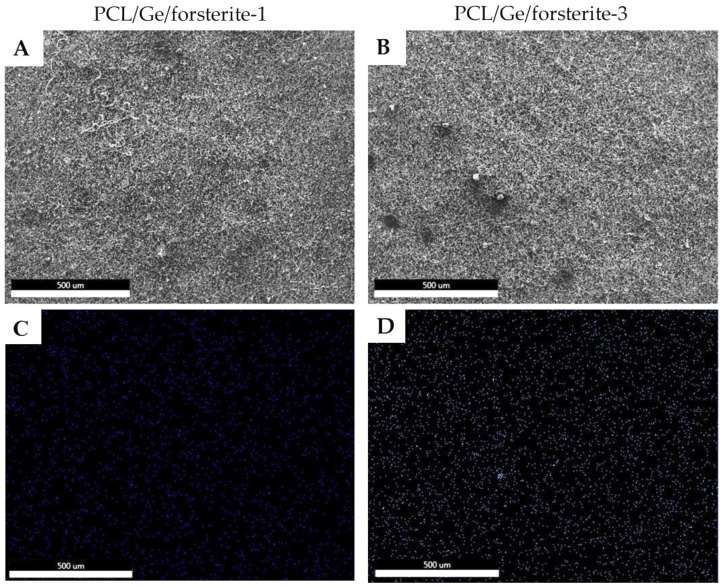
FESEM micrographs (**A**) and (**B**), and the distribution map of Mg element (**C**) and (**D**) of the electrospun PCL/Ge nanofibers with 1% and 3% forsterite nanoparticles.

The morphology and the corresponding EDS analysis of the nanoparticles distribution in the PCL/Ge nanofibers are presented in Figure 2. It can be observed that the nanoparticles are uniformly distributed on the coatings. It can be also discerned that the dispersion of the nanoparticles in the nanofibers with 1 wt.% forsterite is better than the 3 wt.% loaded sample. The low amount of agglomeration in the PCL/Ge/forsterite-3 sample can be attributed to the strong surface energy among the nanoparticles [44]. 

Since the porosity influences the scaffold’s cell adhesion and proliferation, it is essential to consider this scaffold characteristic during the tissue engineering [45]. It was reported that the porosity of the electrospun nanofibers are mostly controlled by the diameter of the nanofibers [46]. The porosity of the samples was measured based on the FESEM micrographs (Figure 1) and reported in Table 2. The porosity of the electrospun scaffolds was reduced by introducing 1 wt.% of nanoparticles and then increased at the nanoparticles content of 3 wt.%. Therefore, the effect of the amount of nanoparticles on the porosity was similar to the fiber diameter. The lowest porosity content was present in the coatings having thinner nanofibers. On the other hand, the highest porosity was assigned to the PCL/Ge/forsterite-3 nanofibers at 82.6% ± 0.2%, which had a thicker fiber diameter. Generally, all of the samples illustrated porosity above 70%, which is apt for medical applications [47]. Therefore, it is anticipated that all of the electrospun mats would have a high potential for cell attachment and proliferation.

**Figure 3:**
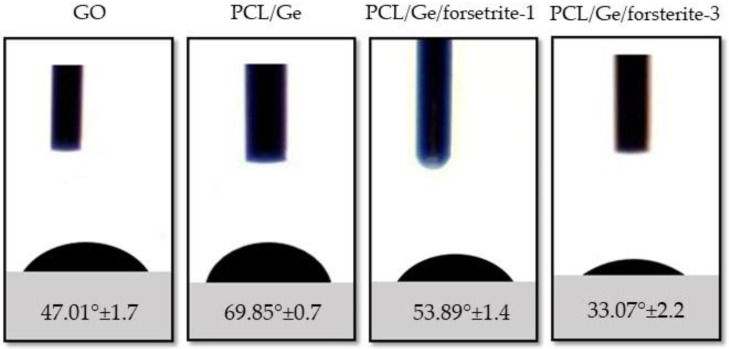
The water contact angles of GO layer and PCL/Ge nanofibers containing 0, 1 and 3 wt.% forsterite.

Figure 3 shows the obtained results of the wettability analysis by measuring the water contact angle for the PCL/Ge, PCL/Ge/forsterite-1 and PCL/Ge/forsterite-3 coatings. The relaxation time of the water droplet was 10 s. According to Sup Kim et al. [48], the contact angle of PCL was reported to be 120°, hence it is clear that the incorporation of gelatin increases the hydrophilicity of PCL nanofibers, which is due to the existence of amine and carboxylic groups in gelatin [49]. As can be seen in Figure 3, when the forsterite nanoparticles content increased from 1 to 3 wt.%, the water contact angle of the nanofibers decreased from 53.59° to 35.55°. Therefore, the hydrophilicity of the nanofibers is affected by the concentration of the nanoparticles. As a result, it is expected that cells would show higher extent adhesion on PCL/Ge/forsterite-3 nanofibers due to increased hydrophilicity.

The weight loss percentage of the samples was measured after 21 days’ soaking in PBS at 37 °C and pH = 7.4. The results are summarized in Table 2. The weight loss of the PCL/Ge nanofibers increased from ca. 12% to ca. 18% by increasing the forsterite content from 0 to 3 wt.%. Therefore, increasing the forsterite content increased the hydrophilicity, porosity and the weight loss of the scaffolds. The degradation of the coating can be associated not only with the hydrolysis of gelatin but also the diffusion of the nanoparticles from the surface of the nanofibrous coating to the solution [50]. Moreover, due to the highly crystalline phase of PCL, its weight loss is considered to be negligible [51]. 

**Figure 4:**
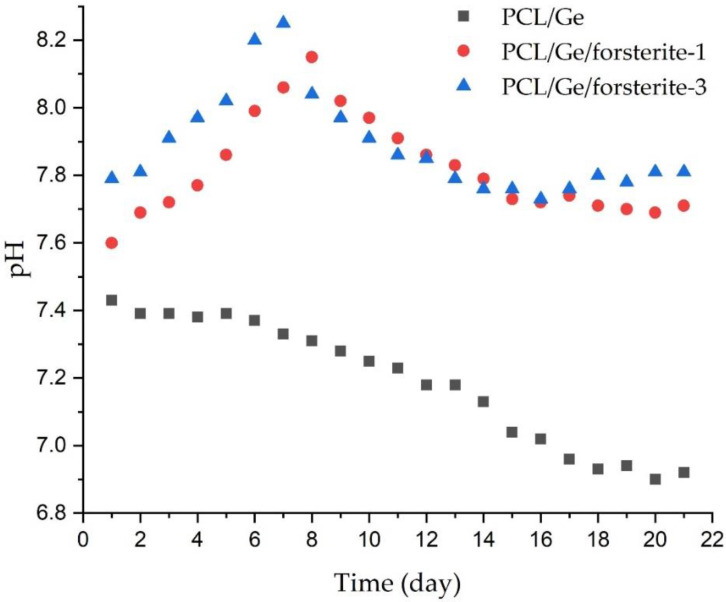
The pH values of the PBS solutions containing PCL/Ge with 0, 1 and 3 wt.% forsterite during 21 days immersion.

The pH changes of the PBS solutions containing the scaffolds were assessed and depicted in Figure 4. It is clear that the pH values of the solution reduced from 7.4 to 6.9 in the PBS solution of the PCL/Ge nanofibers within 21 days. Releasing of acidic products from the degradation of the PCL and gelatin is responsible for the decrease in pH values [52]. In contrast to PCL/Ge, the amount of pH in the solution containing PCL/Ge/forsterite with 1 and 3 wt.% nanoparticles increased to 8.1 and 8.2, respectively, during the first week. The release of the Mg ions from forsterite incorporated into the scaffolds increased the alkalinity of the solution and consequently the pH value [52]. Moreover, the pH slightly decreased in the next week due to polymer degradation and remained constant. 

### 3.2. Bioactivity of the Electrospun Scaffolds

**Figure 5:**
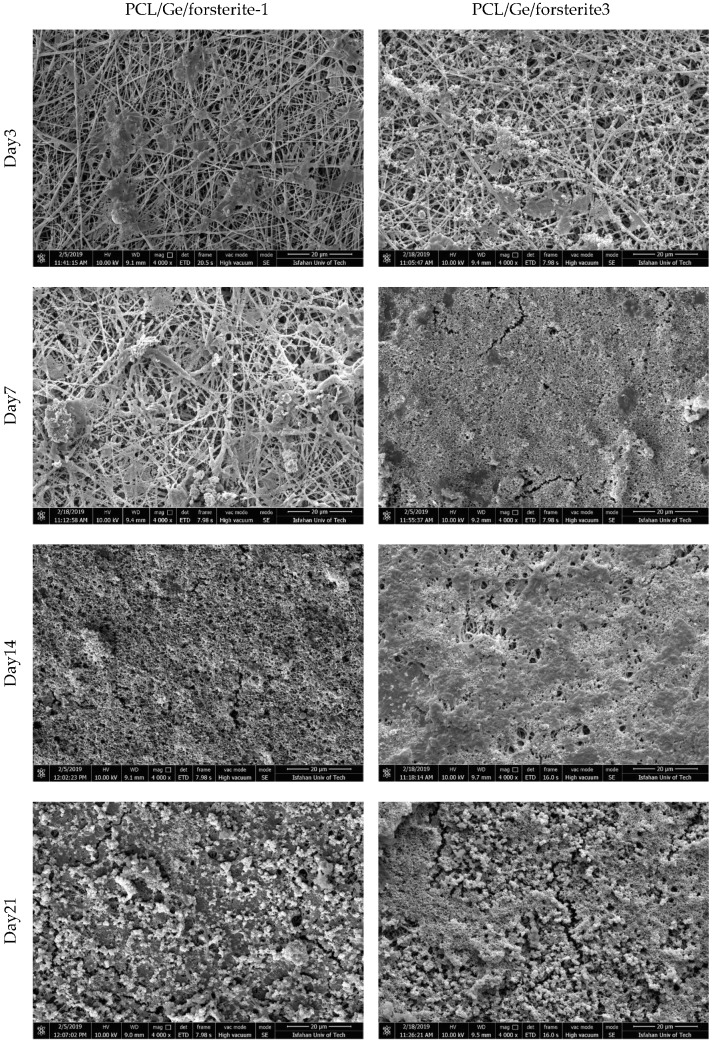
FESEM micrographs of the PCL/Ge/forsterite with 1 and 3 wt.% after 3, 7, 14, and 21 days immersion in the SBF solution.

**Figure 6:**
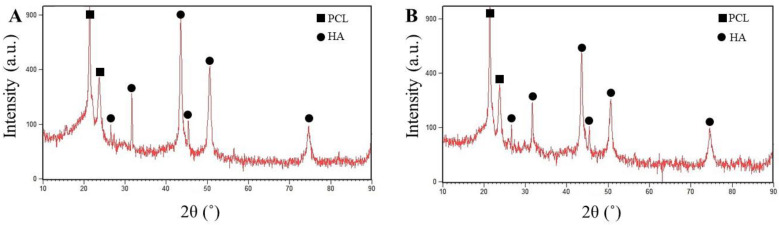
XRD patterns of PCL/Ge nanofibers containing (**A**) 1 and (**B**) 3% of forsterite after 21 days of immersion in SBF.

Osteoconductivity is a crucial characteristic of the implants utilized in bone tissue engineering applications to predict bone regeneration during implant employment [41]. The osteoconductivity of the electrospun nanofibers was evaluated by studying the capability of the coated samples in the creation of bone-like apatite. The PCL/Ge nanofibers containing various concentrations of the nanoparticle (1 and 3 wt.%) were soaked in SBF for 3, 7, 14 and 21 days. Figure 5 demonstrates the FESEM images of the PCL/Ge/forsterite with 1 and 3 wt.% after immersion in SBF. The bone-like apatite deposition was found to form on the surface of the resulting electrospun structure after three days in SBF and noticeably increased within 21 days. The presence of the nanoparticle in the scaffold structure led to the formation of the silanol (–Si–OH) groups, which contributed to Ca-P nucleation. As a result, the interaction of phosphate and carbonate groups in SBF and positively charged positions of the Ca-P nucleation caused phosphate layer formation during the immersion time [53]. From Figure 5, it can be seen that the HA formation on the electrospun structures having 3 wt.% of forsterite is significantly higher than the PCL/Ge/forsterite-1 structure, especially in three and seven days’ immersion. Figure 6 presents the WAXS profiles of the samples PCL/Ge/forsterite-1 and PCL/Ge/forsterite-3 after 21 days soaking in SBF. From the diffractograms, the peaks at 2Ө of 21.4° and 23.8° can be ascribed to the existence of PCL in the structure [26]. In addition, the peaks at 26.7°, 31.7°, 43.6°, 45.5°, 50.7°, and 74.6° can be related to the created HA on the electrospun substrates containing nanoparticles. The observed peaks for HA were also reported for HA in previous works [14,54]. The XRD patterns also confirm the formation of HA after 21 days’ incubation of samples in SBF.

### 3.3. Cell Culture Studies

**Figure 7:**
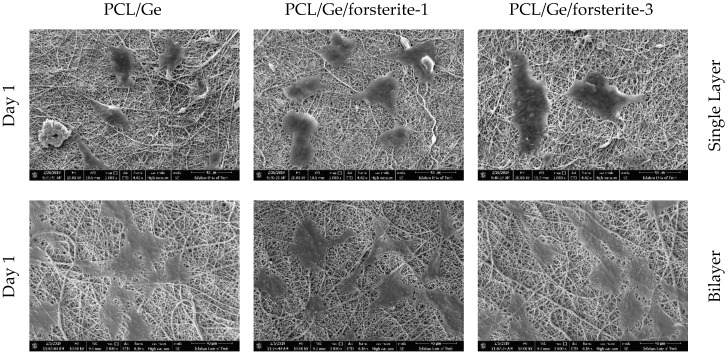

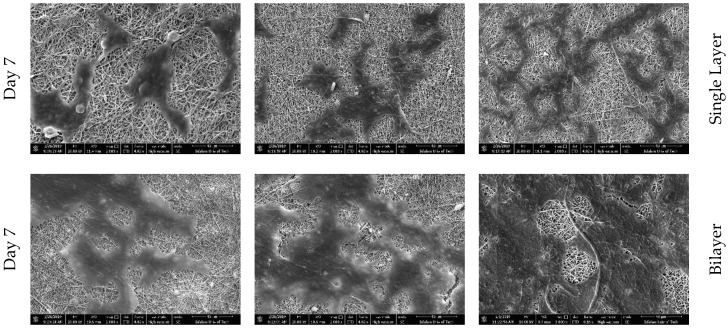
Morphology of the MG-63 cells on PCL/Ge/forsterite nanofibers with 1 and 3 wt.% and GO-PCL/Ge/forsterite with 1 and 3 wt.% after one and seven days of cell culture.

**Figure 8:**
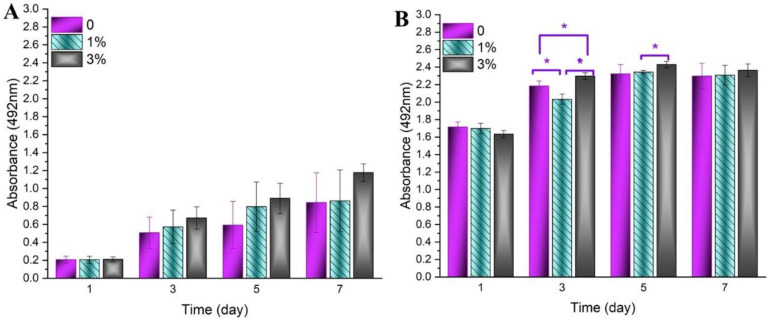
The MTS results of PCL/Ge structures containing 0, 1 and 3 wt.% forsterite nanoparticles, (**A**) without the GO layer and (**B**) with the GO layer (*significant difference at *p_value_* < 0.05).

Cell attachment and proliferation are the results of efficient cell-material interactions [55]. The cell morphology on the PCL/Ge and GO-PCL/Ge electrospun structures containing 1 and 3 wt.% forsterite nanoparticles after one and seven days of MG-63 cells seeding is shown in Figure 7. It can be observed that the cells were well attached and spread on all the samples due to the proper interactions between the cells and the coatings. Specific cellular adhesion and well-spread morphology were higher for the GO-PCL/Ge structure rather than PCL/Ge after seven days. The better performance of the bilayer structure can be attributed to the presence of GO layer (with OH and COOH groups on the surface) and high hydrophilicity of the coatings. The existence of the surface roughness, as the intrinsic property of GO [36], and GO functional groups assisted the serum protein adsorption as well as cell attachment [56]. Additionally, the cells showed better growth on the structures containing a higher amount of the nanoparticles. The MTS results are illustrated in Figure 8 where the progressive growth of the cells confirms the non-cytotoxicity of the coatings [57]. All the bilayer structures with various amounts of the nanoparticles showed effective compatibility and interactions with the cells. The GO-PCL/Ge with 3 wt.% nanoparticles showed a noticeably higher cell viability in comparison with other nanostructures. The higher hydrophilicity of the coatings in the presence of the GO layer and the larger amount of the nanoparticles increased the cell viability. Besides, adsorption of the serum protein is affected by surface oxygen-containing groups of GO as its intrinsic feature [58]. Another reasonable explanation for the good cell growth is the conductivity of GO having oxygenated groups on its structure. Although GO is a poor conductor compared to graphene, it has higher conductivity than PCL/Ge nanofibrous layer [59].

## 4. Conclusions

To recapitulate, a bilayer bioactive coating containing GO layer and nanofibrous PCL/Ge/forsterite was applied on 316LSS to develop a potential platform for bone implant application.

- Characterization of the nanofiber layer revealed the formation of a uniform beadless nanofibrous layer on the surface of the GO layer. It was also indicated that the forsterite nanoparticles were well-distributed on the top layer. The presence of gelatin and forsterite nanoparticles increased the wettability and biodegradation rate of the top layer (electrospun nanofibrous layer) which marks a development in bilayer coating in bone implant applications.
- The bioactivity results indicated the formation of HA on the surface of the nanofiber structures which was subsequently confirmed by XRD. the incorporation of the forsterite nanoparticles increased the bioactivity of the samples, especially after 14 and 21 days of soaking in the SBF solution.
- The PCL/Ge/forsterite and GO-PCL/Ge/forsterite coatings were found to be non-cytotoxic structures with an ability to enhance cell attachment and proliferation. Furthermore, the enhanced adhesion and growth of MG63 cells on bilayer coatings in comparison with nanocomposite coatings revealed the beneficial biocompatibility and hydrophilicity of GO due to functional groups on its surface as well as high surface roughness.

## Figures and Tables

**Table 1:** Physical properties of the solutions.

Nanofiber Composition	Viscosity (cP)	Conductivity (µS/cm)
**PCL/Ge**	910 ± 32	271 ± 13
**PCL/Ge/forsterite-1**	980 ± 24	288 ± 10
**PCL/Ge/forsterite-3**	1400 ± 100	290 ± 20

**Table 2:** Morphology characteristic of the electrospun scaffolds.

Nanofiber Composition	Fiber Diameter (nm)	Porosity (%)	Weight Loss (%)
**PCL/Ge**	167 ± 29	77.4 ± 0.2	12.0 ± 0.2
**PCL/Ge/forsterite-1**	148 ± 36	71.1 ± 0.1	15.0 ± 0.2
**PCL/Ge/forsterite-3**	171 ± 43	82.6 ± 0.2	17.9 ± 0.1
